# Real-world efficacy, safety, and associated biomarkers of cadonilimab in cervical cancer: a prospective observational study

**DOI:** 10.3389/fimmu.2026.1723923

**Published:** 2026-04-27

**Authors:** Xiaotong Lian, Huatao Qi, Dan Lin, Qiong Wu, Yu Lei

**Affiliations:** 1 Guangxi Medical University Affiliated Tumor Hospital, Nanning, Guangxi, China; 2 Guangxi Medical University, Nanning, Guangxi, China; 3 Guangxi Center For Food and Drug Evaluation & Inspection, Nanning, Guangxi, China

**Keywords:** bispecific antibody, cadonilimab, cervical cancer, immune checkpoint inhibitors, PD-1/CTLA-4

## Abstract

**Objective:**

To evaluate the real-world efficacy and safety of cadonilimab (a bispecific antibody targeting PD-1 and CTLA-4) in combination with chemotherapy with or without bevacizumab for cervical cancer and to identify potential biomarkers.

**Methods:**

This preliminary report analyzes the first 51 consecutive patients from a protocol-driven observational cohort initiating cadonilimab (≥2 cycles) between June 2022 and August 2025. The treatment regimens were: cadonilimab + chemotherapy + bevacizumab (n=22), cadonilimab + chemotherapy (n=24), or cadonilimab alone (n=5). Standardized data collection included clinicopathological variables, peripheral blood biomarkers, and protocol-defined tumor assessments (RECIST v1.1 every 6 weeks). Interim efficacy endpoints (objective response rate [ORR], disease control rate [DCR], median progression-free survival [mPFS]) and safety (CTCAE v5.0) were evaluated, with multivariate analyses to identify predictive factors.

**Interim results:**

The median follow-up was 11.0 months. At the first tumor evaluation timepoint after completing two treatment cycles, 15 patients achieved complete response (CR), 22 patients achieved partial response (PR), and 9 patients achieved stable disease (SD), with an ORR of 72.5% and a DCR of 90.2%. At the data cutoff date (December 2025), the median PFS was 7.0 months (IQR: 4.0-10.0) and the DCR was 37.3% (19/51). Multivariable analysis revealed that squamous cell carcinoma histology (OR = 4.471, 95% CI = 1.037–21.699; P = 0.045) and baseline IL-6 levels ≤5.4 pg/mL (OR = 4.494, 95% CI = 1.089–18.541; P = 0.038) were independently associated with higher odds of achieving an objective response. Regarding safety, hematologic toxicities were the most common (74.5%), which may be related to the chemotherapeutic agents used in the combination therapy. Immune-related adverse events (irAEs) included liver function abnormalities in 39.2% of patients and skin and subcutaneous tissue disorders in 23.5% of patients. Analysis of immune-related dermal toxicity identified that a baseline Systemic Immune-Inflammation Index (SII) ≥660 (OR = 8.742, 95% CI = 1.372–55.648, P = 0.022) and CD4^+^PD-1^+^ >42.10% (OR = 18.121, 95% CI = 1.368–239.948, P = 0.028) were independent risk factors, whereas IL-17 >21.4 pg/mL (OR = 0.042, 95% CI = 0.003–0.542, P = 0.015) was an independent protective factor.

**Conclusions:**

In this real-world cohort, cadonilimab showed promising early efficacy and a manageable safety profile in cervical cancer. Identified biomarkers for response and immune-related dermal toxicity require validation in larger, prospective studies with longer follow-up.

## 1 Introduction

Cervical cancer remains a major global health challenge. Latest global cancer statistics reveal that approximately 660,000 new cervical cancer cases and 350,000 deaths were reported worldwide in 2022, establishing cervical cancer as the fourth most commonly diagnosed cancer and fourth leading cause of cancer-related mortality among women (1). The burden of cervical cancer is particularly pronounced in developing countries (2, 3): over 70% of cases are diagnosed at an advanced stage, with recurrence and metastasis rates reaching 15%–61% within the first year after treatment, and the 5-year survival rate is only 17%.

In recent years, advances in immunotherapy, targeted therapy, radiotherapy, and surgical techniques have significantly transformed the treatment landscape for cervical cancer. Among these, immune checkpoint inhibitors (ICIs), which activate the host immune system to target tumor cells, have demonstrated remarkable efficacy across various solid tumors and have become a cornerstone strategy in oncology. In 2018, pembrolizumab was approved for platinum-resistant cervical cancer; subsequently, the landmark KEYNOTE-826 trial established pembrolizumab combined with chemotherapy (with or without bevacizumab) as the first-line standard of care for persistent, recurrent, or metastatic cervical cancer (4). Despite these advances, significant unmet clinical needs remain, particularly for patients who progress on or are ineligible for first-line pembrolizumab-based therapy, as well as those with low PD-L1 expression and poor response to PD-1 monotherapy.

Simultaneous blockade of the PD-1 and CTLA-4 signaling pathways has been proposed as a potential strategy to overcome resistance and enhance anti-tumor immunity beyond that achieved with PD-1 inhibition alone. CTLA-4 and PD-1 exert complementary yet non-redundant inhibitory effects on T-cell function. Combined blockade has demonstrated synergistic efficacy and prolonged survival in multiple metastatic solid tumors. For example, in a phase II trial evaluating balstilimab (anti-PD-1) plus zalifrelimab (anti-CTLA-4) as second-line therapy for recurrent/metastatic cervical cancer (R/M CC), the confirmed objective response rate (ORR) was 25.6% (4, 5). The CheckMate-358 trial further showed that nivolumab (anti-PD-1) combined with ipilimumab (anti-CTLA-4) induced durable and clinically meaningful responses in patients with R/M CC (6).

Cadonilimab is a novel bispecific antibody targeting both PD-1 and CTLA-4. It has shown robust anti-tumor activity in early clinical studies and was approved by the China National Medical Products Administration (NMPA) in June 2022 for second-line treatment. The phase III COMPASSION-16 study demonstrated that adding cadonilimab to standard therapy significantly improved progression-free survival (PFS) and overall survival (OS) in patients with advanced cervical cancer in the first-line setting (7). In June 2022, based on this study, cadonilimab was officially approved in China for the second-line treatment of platinum-resistant cervical cancer (8). A recent real-world study (N = 101) further reported post-marketing clinical experience with this regimen (9).

Although cadonilimab has shown a significantly higher objective response rate compared to PD-1/PD-L1 inhibitors and even some combination therapies in previously treated recurrent or metastatic cervical cancer, with a relatively low incidence of grade ≥3 immune-related adverse events (irAEs) and better tolerability, attention should be paid to its relatively higher incidence of specific adverse events such as anemia and elevated transaminases. The potential synergistic toxicity risk associated with dual-target inhibition, although low in incidence, necessitates heightened vigilance in clinical use, especially when combined with chemotherapy or other agents. Furthermore, as a relatively new therapeutic agent, its full spectrum of rare adverse events, delayed toxicities (e.g., occurring months after treatment cessation), and long-term safety profile with extended use (>2 years) require further real-world evidence.

Therefore, we conducted this prospective observational cohort study to systematically evaluate the real-world efficacy and safety of cadonilimab in patients with cervical cancer, analyze prognostic factors across different subgroups, and provide actionable insights to optimize clinical management and improve patient outcomes.

## 2 Methods

### 2.1 Data collection

This ongoing observational study evaluates adult patients with histopathologically confirmed cervical cancer who initiated cadonilimab treatment at Guangxi Medical University Affiliated Tumor Hospital from June 2022, with follow-up ongoing through December 2025. Eligible patients were required to have experienced disease progression following, or intolerance to, at least one prior line of systemic therapy, which could include anti-PD-1 antibody therapy, regardless of PD-L1 expression status. Patients were required to have at least one measurable lesion after prior therapy, adequate organ function, and an Eastern Cooperative Oncology Group (ECOG) performance status of 0 or 1. Those with significant autoimmune diseases were excluded. All baseline data, including clinicopathological characteristics and biomarker profiles, were systematically collected within one week prior to the initiation of cadonilimab. To ensure the validity and credibility of the study results (10, 11), we required that the overall missingness of baseline data for each included patient be <10%, and that the missing rate for any single baseline characteristic across the total sample also not exceed 10%. Patients with more than 10% missing baseline data were excluded from the analysis. Additionally, patients who could not adhere to the treatment and/or follow-up protocol were also excluded, defined as those who did not receive at least two cycles of cadonilimab treatment and complete at least one post-baseline tumor assessment. Clinical data were extracted from medical records and follow-up visits. The study was approved by the Institutional Ethics Committee (Guangxi Medical University Affiliated Tumor Hospital) and conducted in accordance with the Declaration of Helsinki. All participants provided written informed consent.

### 2.2 Study protocol implementation

Prospective data capture: Electronic case report forms (eCRFs) were completed at each treatment cycle to ensure standardized and real-time data collection.

Protocol-defined assessments: Tumor response was evaluated by contrast-enhanced CT/MRI every 6 weeks (± 7 days), with centralized RECIST v1.1 adjudication by an independent imaging review committee.

Active safety monitoring: Adverse events (AEs) were graded per CTCAE v5.0 at all visits, with real-time reporting to the safety monitoring team for immediate intervention if needed.

### 2.3 Treatment protocol and assessments

Patients received intravenous cadonilimab (10 mg/kg) every 3 weeks until disease progression, unacceptable toxicity, or treatment discontinuation. The choice of treatment regimen (cadonilimab ± chemotherapy ± bevacizumab) was determined by the treating physicians based on patients’ baseline organ function, performance status, and clinical judgment. Tumor response was evaluated every 2 cycles per RECIST v1.1 criteria, classifying outcomes as complete response (CR), partial response (PR), stable disease (SD), or progressive disease (PD). Suspected PD required confirmatory imaging ≥4 weeks later. The primary endpoint was objective response rate (ORR, CR+PR); secondary endpoints included disease control rate (DCR, CR+PR+SD), progression-free survival (PFS; treatment initiation to progression/death), overall survival (OS; treatment initiation to death), 3-month progression-free survival rate, and safety (CTCAE v5.0).

### 2.4 Biomarker measurements

Peripheral blood samples were collected from all patients within one week prior to treatment initiation. Complete blood count, lymphocyte subsets (including CD3^+^, CD4^+^, CD8^+^ T cells, B cells, and NK cells), serum immunoglobulins (IgG, IgA, IgM), complement components (C3, C4), and cytokines (IL-6, IL-17, TNF-α) were measured at the institutional clinical laboratory using standard flow cytometry (Navios, Beckman Coulter), immunoturbidimetry (AU5800, Beckman Coulter), and multiplex immunoassay (RaiseCare, Qingdao RaiseCare Biological Technology) according to the manufacturers’ instructions. The Systemic Immune-Inflammation Index (SII) was calculated as (platelet count × neutrophil count)/lymphocyte count.

All biomarker cutoff values used in the analyses were based on the institutional laboratory’s clinical reference range.

### 2.5 Statistical analysis

Given the ongoing nature of this study, we present interim analyses using data current through December 2025. Continuous variables were reported as median (range) and categorical variables as frequency (%). Treatment responses were compared using χ² or Fisher’s exact tests. Survival analyses utilized Kaplan-Meier estimates with log-rank tests. To explore potential predictors of response and toxicity, multivariable logistic regression was used. All analyses involving biomarkers were pre-specified in the statistical analysis plan as exploratory to generate hypotheses for future validation, and no adjustments for multiple comparisons were made. Analyses were performed using SPSS v25.0 and R v4.2.2. P values <0.05 were considered statistically significant.

## 3 Results

### 3.1 Patient characteristics

**Figure 1:**
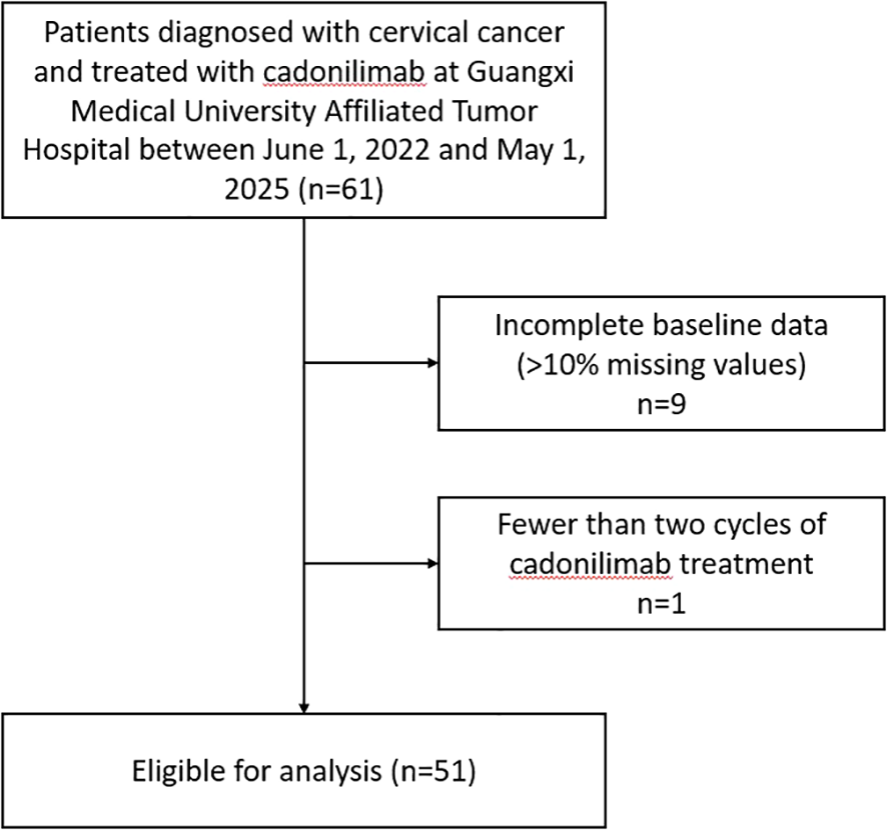
Patient enrollment and selection flowchart: interim analysis cohort.

The cutoff date for data analysis was 31 December 2025. As of the data cutoff, this interim analysis includes 51 protocol-eligible patients who had completed at least two cycles of cadonilimab treatment and at least one post-treatment assessment (**Figure 1**). The completeness of baseline clinical characteristics and laboratory data was high, with an overall missing rate of less than 5% across all assessed variables. The primary source of missing data was PD-L1 expression status, which was unknown for 33 patients (64.7%) due to either lack of initial assessment or unavailable tissue samples for retesting; therefore, PD-L1 expression status was not included in subsequent analyses. This low level of missingness for other key variables ensures a robust foundation for statistical analysis.

The age of patients ranged from 33 to 78 years (median 54 years). Histologically, 39 patients (76.5%) had squamous cell carcinoma, 10 (19.6%) had adenocarcinoma, and 1 (2.0%) had adenosquamous carcinoma. Regarding PD-L1 status, 8 patients (15.7%) were positive, 10 (19.6%) were negative, and the status was unknown for the remaining 33 patients (64.7%) due to either lack of assessment or unavailable tissue samples. In their primary treatment, 20 patients (39.2%) had undergone surgery, and 27 (52.9%) had received definitive radiotherapy, including 9 who received postoperative radiotherapy. 29 patients (56.9%) had received taxane plus platinum chemotherapy as first-line treatment, among whom 11 (21.6%) had also received a prior PD-1 inhibitor. The baseline demographic and clinical characteristics of the patients are presented in **Table 1**. Corresponding laboratory data are presented in **Table 2**.

**Table 1:** Clinical and treatment characteristics at baseline.

Characteristic	Patients (n=51)(%)
Median age (range), years	54 (33-78)
≥65 years	6 (11.8)
Ethnicity	
Han ethnicity	38 (74.5)
Ethnic minorities	13 (25.5)
BMI	
≤23.9 kg/m^2^	36 (70.6)
>23.9 kg/m^2^	15 (29.4)
Menstruation	
Postmenopausal	39 (76.5)
Non-menopausal	12 (23.5)
History of abortion	
Yes	41 (80.4)
No	10 (19.6)
ECOG PS	
0	40 (78.4)
1	11 (21.6)
Pathological diagnosis	
Squamous cell carcinoma	39 (76.5)
Adenocarcinoma	10 (19.6)
Adenosquamous carcinoma	1 (2.0)
FIGO stage	
<III	14 (27.5)
≥III	35 (68.6)
Unknown	2 (3.9)
Metastatic disease	
Yes	40 (78.4)
No	11 (21.6)
Previous therapy	
Surgery	20 (39.2)
Radiotherapy	27 (52.9)
Surgery and radiotherapy	9 (17.6)
Chemotherapy	51 (100.0)
Prior lines of therapy	
First line	34 (66.7)
Second-line or more	17 (33.3)
Prior antineoplastic agents	
Taxane-Platinum	29 (56.9)
PD-1 inhibitors	11 (21.6)
Concomitant treatments during cadonilimab therapy	
Combination with chemotherapy and bevacizumab	22 (43.1)
Cadonilimab plus chemotherapy alone	24 (47.1)
Cadonilimab only	5 (9.8)

**Table 2:** Laboratory parameters at baseline.

Laboratory parameters	Patients (n=51) (%)
PLR (platelet to lymphocyte count ratio)	
<300	29 (56.9)
≥300	22 (43.1)
NLR (neutrophil to lymphocyte ratio)	
<5.0	27 (52.9)
≥5.0	24 (47.1)
SII (Systemic Immune-Inflammation Index)	
<600	7 (13.7)
≥600	44 (86.3)
IL-6	
≤5.4 pg/mL	23 (45.1)
>5.4 pg/mL	28 (54.9)
IL-17	
≤21.4 pg/mL	44 (86.3)
>21.4 pg/mL	7 (13.7)
TNF-α	
≤16.5 pg/mL	49 (96.1)
>16.5 pg/mL	2 (3.9)
Erythrocyte sedimentation rate (ESR)	
<20 mm/h	22 (43.1)
≥20 mm/h	29 (56.9)
C-reactive protein (CRP)	
<10 mg/L	37 (72.5)
≥10 mg/L	14 (27.5)
CD3^+^	
<61%	7 (13.7)
61%-85%	43 (84.3)
>85%	1 (2.0)
CD3^+^CD4^+^	
<28%	10 (19.6)
28%-58%	41 (80.4)
>58%	0 (0.0)
CD3^+^CD8^+^	
<19%	4 (7.8)
19%-48%	46 (90.2)
>48%	1 (2.0)
CD3^+^CD4^+^/CD3^+^CD8^+^	
<0.9	6 (11.8)
0.9-3.6	44 (86.3)
>3.6	1 (2.0)
CD3^-^CD56^+^	
<10%	7 (13.7)
10%-25%	40 (78.4)
>25%	4 (7.8)
CD19^+^	
<7%	4 (7.8)
7%-23%	44 (86.4)
>23%	3 (5.9)
Complement C3	
<0.90 g/L	5 (9.8)
0.90 g/L-1.50 g/L	43 (84.3)
>1.50 g/L	3 (5.9)
Complement C4	
<0.20 g/L	3 (5.9)
0.20 g/L-0.40 g/L	46 (90.2)
>0.40 g/L	2 (3.9)
IgG	
<8 g/L	0 (0.0)
8 g/L-16 g/L	46 (90.2)
>16 g/L	5 (9.8)
IgA	
<0.7 g/L	0 (0.0)
0.7 g/L-4.0 g/L	48 (94.1)
>4.0 g/L	3 (5.9)
IgM	
<0.35 g/L	0 (0.0)
0.35 g/L-2.5 g/L	51 (100.0)
>2.5 g/L	0 (0.0)
CD3^+^CD28^+^	
<32.13%	1 (2.0)
32.13%-79.33%	45 (88.2)
>79.33%	5 (9.8)
CD3^+^PD-1^+^	
≤40.82%	39 (86.5)
>40.82%	12 (23.5)
CD4^+^PD-1^+^	
≤42.10%	35 (68.6)
>42.10%	16 (31.4)
CD8^+^CD28^+^	
<14.55%	7 (13.7)
14.55%-73.11%	44 (86.3)
>73.11%	0 (0.0)
CD8^+^PD-1^+^	
≤41%	41 (80.4)
>41%	10 (19.6)
CD4^+^CD28^+^	
<48.93%	1 (2.0)
≥48.93%	50 (98.0)

Cadonilimab was given at 10 mg/kg intravenously every three weeks according to the assessment of patients’ baseline status and economic factors. Among the 51 patients, 22 (43.1%) received cadonilimab in combination with chemotherapy and bevacizumab; 24 (47.1%) received cadonilimab plus chemotherapy alone, limited by their baseline hepatic or renal function; and 5 (9.8%) received cadonilimab monotherapy due to intolerance to chemotherapy-related side effects.

In summary, all patients in this cohort received cadonilimab as second-line or later therapy, having progressed after at least one prior systemic treatment.

### 3.2 Efficacy

**Figure 2:**
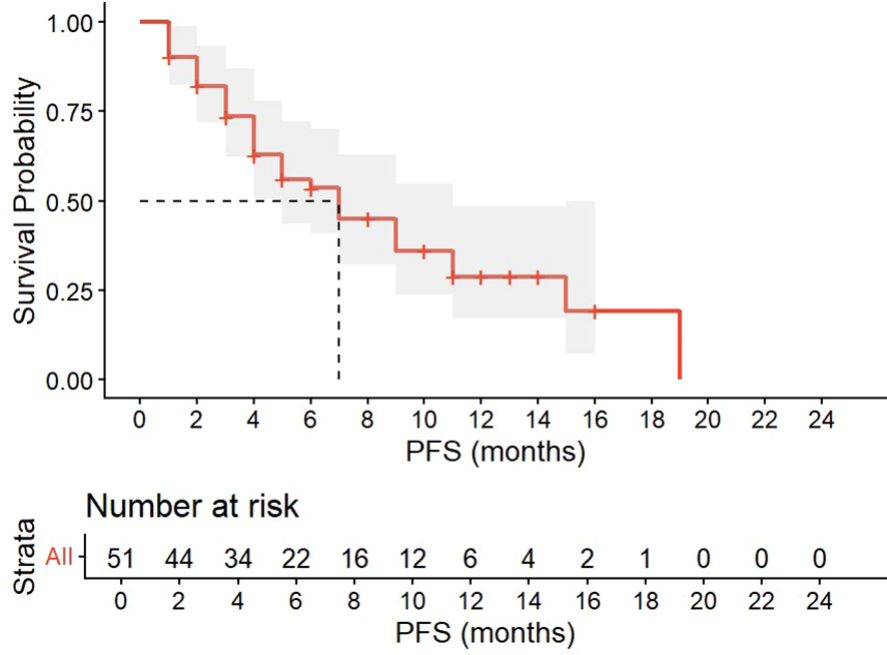
Kaplan–Meier survival curves of PFS in the patients treated with cadonilimab.

The median follow-up for the cohort was 11.0 months (IQR: 8.6–13.4). At the first tumor evaluation timepoint after completing two treatment cycles, 15 patients achieved a CR, 22 patients showed a PR, and 9 patients had an SD, with an ORR of 72.5% and a DCR of 90.2%. When examining outcomes by treatment regimen, the ORR was 72.7% (16/22) in the triple-therapy group (cadonilimab + chemotherapy + bevacizumab), 70.8% (17/24) in the double-therapy group (cadonilimab + chemotherapy), and 80.0% (4/5) in the monotherapy group. At the final follow-up cutoff of December 31, 2025, 32 patients had experienced PD, with 3 deaths attributed to progression. The DCR was 37.3% (19/51). The median progression-free survival (mPFS) was 7.0 months (IQR: 4.0–10.0) (**Figure 2**). The median overall survival (mOS) was not reached. Collectively, cadonilimab showed promising early efficacy, although longer follow-up is needed to evaluate the durability of response.

### 3.3 Safety

**Figure 3:**
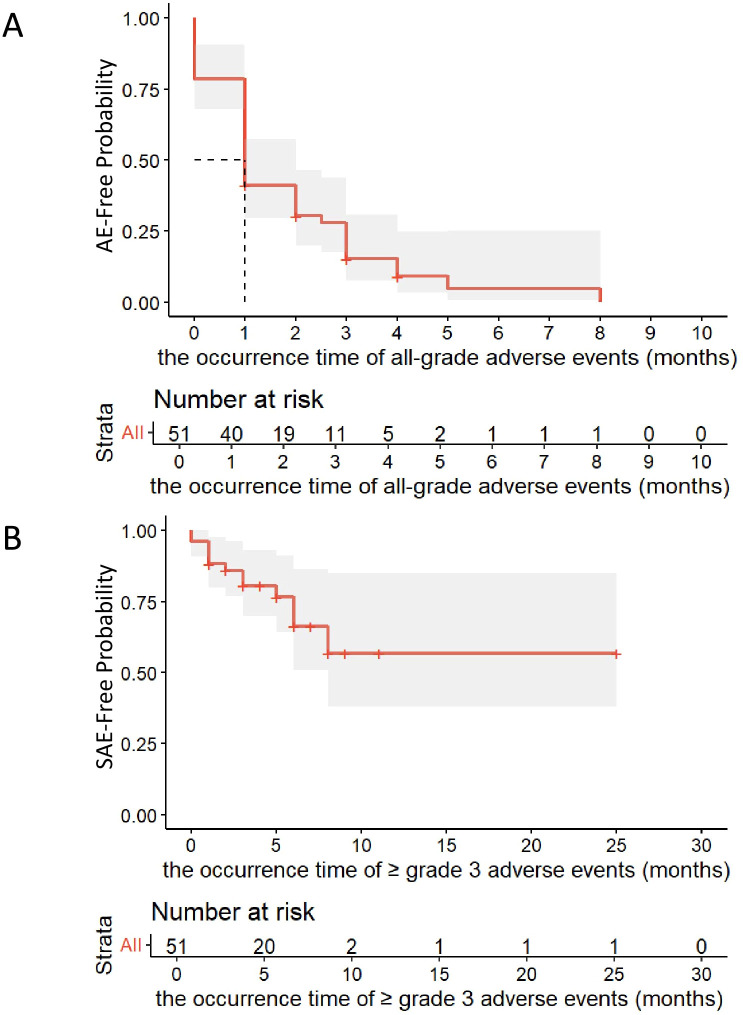
Kaplan-Meier curves for the time to first onset of adverse events. **(A)** All-grade adverse events. **(B)** ≥ Grade 3 adverse events.

**Table 3** presents the adverse events observed in all patients treated with cadonilimab. **Figure 3A** and **Figure 3B** display the Kaplan-Meier curves for the time to first onset of all-grade adverse events and grade 3 or higher adverse events, respectively. Among the 51 treated participants, 46 (90.2%) experienced treatment-emergent adverse events, with a median time to first all-grade adverse event of 1.0 month (95% CI: 0.6–1.4); 16 participants (31.4%) developed grade 3 or higher adverse events, with a median time to first G3+ adverse event of 8.0 months (95% CI: not estimable by the Kaplan-Meier method). In terms of system organ class, hematological toxicities were the most common, occurring in 38 patients (74.5%), followed by liver function-related events in 20 patients (39.2%), and immune-related skin and subcutaneous tissue disorders in 12 patients (23.5%). Among these, anemia and decreased white blood cell count were the most common adverse events across all grades as well as grade 3 or higher. Adverse events involving other organ systems in patients receiving cadonilimab were predominantly mild (grade 1-2) and generally manageable.

**Table 3:** Adverse events in total patients.

Adverse events	Any grade	Grade 3–5
Any treatment-emergent adverse events	46 (90.2)	16 (31.4)
Hematological adverse events	38 (74.5)	13 (25.5)
White blood cell count decreased	26 (51.0)	10 (19.6)
Red blood cell count decreased	17 (33.3)	7 (13.7)
Anaemia	35 (68.6)	11 (21.6)
Platelet count decreased	10 (19.6)	6 (11.8)
Neutrophil count decreased	6 (11.8)	4 (7.8)
Skin and subcutaneous tissue disorders	12 (23.5)	4 (7.8)
Rash	9 (17.6)	4 (7.8)
Pruritus	5 (9.8)	0 (0.0)
hypersensitivity reactions	9 (17.6)	1 (2.0)
Infusion-related reaction	3 (5.9)	1 (2.0)
Nausea	0 (0.0)	0 (0.0)
Vomiting	1 (2.0)	0 (0.0)
Decreased appetite	4 (7.8)	3 (5.9)
Pain in the extremity	3 (5.9)	2 (3.9)
Hypothyroidism	1 (2.0)	0 (0.0)
Hyperthyroidism	0 (0.0)	0 (0.0)
Liver function-related adverse events	20 (39.2)	0 (0.0)
Alanine aminotransferase increased	18 (35.3)	0 (0.0)
Aspartate aminotransferase increased	19 (37.3)	0 (0.0)
Hepatic impairment	14 (27.5)	0 (0.0)
Others	0 (0.0)	0 (0.0)

### 3.4 Predictive biomarkers

To explore potential clinical factors that may enhance the efficacy of cadonilimab, **Table 4** presents the association between patient clinical characteristics and the ORR. Multivariate analysis identified two independent factors associated with objective response rate: squamous cell carcinoma histology (OR = 4.471, 95% CI = 1.037–21.699, P = 0.045) and low baseline IL-6 levels (OR = 4.494, 95% CI = 1.089–18.541, P = 0.038). Additionally, ethnic minority status and low baseline CD3^+^ levels showed a trend toward higher ORR in univariate logistic regression analysis, though without statistical significance, warranting further validation through long-term follow-up.

**Table 4:** Logistic regression analysis of clinical factors on ORR.

Characteristic	Univariate analysis			Multivariate analysis		
	P value	OR	95% CI	P value	OR	95% CI
Age (≥65 vs. <65)	0.203	3.091	0.543-17.588			
Ethnicity (Han vs. minorities)	0.088	0.311	0.081-1.189			
BMI (>23.9 vs. ≤23.9)	0.583	1.444	0.388-5.376			
Postmenopausal	0.603	0.690	0.170-2.794			
History of abortion	0.840	0.856	0.187-3.907			
ECOG PS (0 vs. 1)	0.154	0.208	0.024-1.800			
Pathological diagnosis (Squamous cell carcinoma vs. Non-squamous cell carcinoma)	0.034	4.650	1.125-19.212	0.045	4.741	1.037-21.699
FIGO stage (≥IIIvs. <III)	0.486	0.623	0.165-2.357			
Metastatic disease	0.140	0.348	0.086-1.412			
Previous therapy						
Surgery	0.335	1.846	0.531-6.420			
Radiotherapy	0.111	2.941	0.780-11.094			
Lines of therapy (first line vs. others)	0.378	1.773	0.497-6.324			
Prior antineoplastic agents						
Taxane-Platinum	0.512	1.530	0.430-5.449			
PD-1 inhibitors	0.988	0.989	0.221-4.419			
PLR (≥300 vs. <300)	0.203	0.422	0.112-1.591			
NLR (≥5.0 vs. <5.0)	0.796	1.176	0.343-4.029			
SII (≥600 vs. <600)	0.999	753888260.0	0.000-			
IL-6 (>5.4 vs. ≤5.4)	0.025	4.615	1.207-17.656	0.038	4.494	1.089-18.541
IL-17 (>21.4 vs. ≤21.4)	0.943	1.067	0.182-6.256			
TNF-α(>16.5 vs. ≤16.5)	0.999	0.000	0.000-			
ESR (≥20 vs. <20)	0.512	1.530	0.430-5.449			
CRP (≥10 vs. <10)	0.912	1.080	0.275-4.241			
CD3^+^ (<61 vs. ≥61)	0.073	4.533	0.867-23.716			
CD3^+^CD4^+^ (<28 vs. ≥28)	0.327	0.484	0.113-2.067			
CD3^+^CD8^+^ (<19 vs. ≥19)	0.310	2.917	0.369-23.038			
CD3^+^CD4^+^/CD3^+^CD8^+^ (<0.9 vs. ≥0.9)	0.732	1.375	0.222-8.496			
CD3^-^CD56^+^ (<10 vs. ≥10)	0.943	1.067	0.182-6.256			
CD19^+^ (>23 vs. ≤23)	0.158	6.000	0.499-72.207			
C3 (<0.90 vs. ≥0.90)	0.513	1.889	0.281-12.710			
C4 (<0.20 vs ≥0.20)	0.999	0.000	0.000-			
IgG (>16 vs. ≤16)	0.999	0.000	0.000-			
IgA (>4.0 vs. ≤4.0)	0.815	1.346	0.112-16.130			
CD3^+^CD28^+^ (>79.33 vs. ≤79.33)	0.513	1.889	0.281-12.710			
CD3^+^PD-1^+^ (>40.82 vs. ≤40.82)	0.999	0.000	0.000-			
CD4^+^PD-1^+^ (>42.10 vs. ≤42.10)	0.121	0.274	0.053-1.408			
CD8^+^CD28^+^ (<14.55 vs. ≥14.55)	0.414	2.516	0.275-23.025			
CD8^+^PD-1^+^ (>41 vs. ≤41)	0.999	0.000	0.000-			
CD4^+^CD28^+^ (≥48.93 vs. <48.93)	1.000	0.000	0.000-			
Concomitant treatments during cadonilimab therapy						
(Chemo) radiotherapy	0.943	0.938	0.160-5.498			
Bevacizumab	0.488	1.562	0.442-5.523			

*The multivariate Logistic regression analysis was performed using the backward likelihood ratio (LR) method.

We next examined factors associated with adverse events. Baseline complement C3 levels <0.90 g/L (OR = 9.556, 95% CI = 1.127–81.054, P = 0.039) were significantly correlated with the occurrence of adverse events. A CD3^+^CD4^+^/CD3^+^CD8^+^ ratio <0.9 showed a similar but non-significant trend (OR = 7.000, 95% CI = 0.890–55.048, P = 0.064) (**Table 5**).

**Table 5:** Logistic regression analysis of clinical factors on AEs.

Characteristic	P value	OR	95% CI
Age (≥65 vs. <65)	0.999	0.000	0.000-
Ethnicity (Han vs. minorities)	0.768	1.412	0.143-13.913
BMI (>23.9 vs. ≤23.9)	0.611	0.554	0.057-5.414
Postmenopausal	0.845	1.257	0.127-12.460
History of abortion	0.245	0.316	0.045-2.208
ECOG PS (0 vs. 1)	0.928	0.900	0.090-8.982
Pathological diagnosis (Squamous cell carcinoma vs. Non-squamous cell carcinoma)	0.909	0.875	0.088-8.738
FIGO stage (≥III vs. <III)	0.555	0.563	0.083-3.792
Metastatic disease	0.999	230782120.4	0.000-
Previous therapy			
Surgery	0.329	2.559	0.388-16.882
Radiotherapy	0.546	0.560	0.085-3.673
Lines of therapy (first line vs. others)	0.203	3.429	0.515-22.837
Prior antineoplastic agents			
Taxane-Platinum	0.998	0.000	0.000-
PD-1 inhibitors	0.997	1346229036	0.000-
PLR (≥300 vs. <300)	0.882	0.867	0.132-5.690
NLR (≥5.0 vs. <5.0)	0.231	0.250	0.026-2.411
SII (≥600 vs. <600)	0.999	207112159.3	0.000-
IL-6 (>5.4 vs. ≤5.4)	0.810	0.794	0.121-5.206
IL-17 (>21.4 vs. ≤21.4)	0.671	1.677	0.158-17.534
TNF-α(>16.5 vs. ≤16.5)	0.999	0.000	0.000-
ESR (≥20 vs. <20)	0.998	336557258.9	0.000-
CRP (≥10 vs. <10)	0.513	1.889	0.281-12.710
CD3^+^ (<61 vs. ≥61)	0.671	1.667	0.158-17.534
CD3^+^CD4^+^ (<28 vs. ≥28)	0.245	0.316	0.045-2.208
CD3^+^CD8^+^ (<19 vs. ≥19)	0.999	0.000	0.000-
CD3^+^CD4^+^/CD3^+^CD8^+^ (<0.9 vs. ≥0.9)	0.064	7.000	0.890-55.048
CD3^-^CD56^+^ (<10 vs. ≥10)	0.099	5.467	0.728-41.034
CD19^+^ (>23 vs. ≤23)	0.200	5.500	0.405-74.763
C3 (<0.90 vs. ≥0.90)	0.039	9.556	1.127-81.054
C4 (<0.20 vs ≥0.20)	0.200	5.500	0.405-74.763
IgG (>16 vs. ≤16)	0.434	2.625	0.234-29.504
IgA (>4.0 vs. ≤4.0)	0.200	5.500	0.405-74.763
CD3^+^CD28^+^ (>79.33 vs. ≤79.33)	0.999	0.000	0.000-
CD3^+^PD-1^+^ (>40.82 vs. ≤40.82)	0.372	2.400	0.351-16.394
CD4^+^PD-1^+^ (>42.10 vs. ≤42.10)	0.663	1.524	0.229-10.150
CD8^+^CD28^+^ (<14.55 vs. ≥14.55)	0.671	0.600	0.057-6.312
CD8^+^PD-1^+^ (>41 vs. ≤41)	0.245	3.167	0.453-22.143
CD4^+^CD28^+^ (≥48.93 vs. <48.93)	1.000	179497204.8	0.000-
Concomitant treatments during cadonilimab therapy			
(Chemo) radiotherapy	0.671	0.600	0.057-6.312
Bevacizumab	0.462	0.426	0.044-4.135

Further analysis focusing specifically on immune-related adverse events (irAEs) of the skin and subcutaneous tissue identified distinct predictive markers (**Table 6**). A baseline Systemic Immune-Inflammation Index (SII) ≥660 (OR = 8.742, 95% CI = 1.372–55.648, P = 0.022) and CD4^+^PD-1^+^ >42.10% (OR = 18.121, 95% CI = 1.368–239.948, P = 0.028) were independent risk factors. In contrast, elevated baseline IL-17 (>21.4 pg/mL) was an independent protective factor (OR = 0.042, 95% CI = 0.003–0.542, P = 0.015).

**Table 6:** Logistic regression analysis of clinical factors on immune-related skin and subcutaneous tissue disorders.

Characteristic	Univariate analysis			Multivariate analysis		
	P value	OR	95% CI	P value	OR	95% CI
Previous therapy						
Surgery	0.634	1.391	0.357-5.417			
Radiotherapy	0.128	2.875	0.738-11.194			
Lines of therapy (first line vs. others)	0.176	3.125	0.600-16.265			
Prior antineoplastic agents						
Taxane-Platinum	0.230	2.240	0.600-8.356			
PD-1 inhibitors	0.999	692346420.2	0.000-			
PLR (≥300 vs. <300)	0.157	2.850	0.669-12.146			
NLR (≥5.0 vs. <5.0)	0.090	3.500	0.821-14.927			
SII (≥660 vs. <660)	0.063	3.929	0.931-16.582	0.028	8.742	1.373-55.648
IL-6 (>5.4 vs. ≤5.4)	0.120	3.158	0.741-13.458			
IL-17 (>21.4 vs. ≤21.4)	0.037	0.167	0.031-0.896	0.015	0.042	0.003-0.542
TNF-α(>16.5 vs. ≤16.5)	0.999	0.000	0.000-			
ESR (≥20 vs. <20)	0.230	2.240	0.600-8.356			
CRP (≥10 vs. <10)	0.828	1.179	0.268	5.183		
CD3^+^ (<61 vs. ≥61)	1.000	0.000	0.000-			
CD3^+^CD4^+^ (<28 vs. ≥28)	0.999	0.000	0.000-			
CD3^+^CD8^+^ (<19 vs. ≥19)	0.999	553877295.9	0.000-			
CD3^+^CD4^+^/CD3^+^CD8^+^ (<0.9 vs. ≥0.9)	1.000	510149817.9	0.000-			
CD3^-^CD56^+^ (<10 vs. ≥10)	0.999	553877017.5	0.000-			
CD19^+^ (>23 vs. ≤23)	0.999	538491476.4	0.000-			
C3 (<0.90 vs. ≥0.90)	0.999	570167720.7	0.000-			
C4 (<0.20 vs ≥0.20)	0.999	538491718.7	0.000-			
IgG (>16 vs. ≤16)	0.845	1.257	0.127-12.460			
IgA (>4.0 vs. ≤4.0)	0.683	0.595	0.049-7.193			
CD3^+^CD28^+^ (>79.33 vs. ≤79.33)	0.372	0.417	0.061-2.846			
CD3^+^PD-1^+^ (>40.82 vs. ≤40.82)	0.185	4.321	0.497-37.572			
CD4^+^PD-1^+^ (>42.10 vs. ≤42.10)	0.078	6.875	0.804-58.806	0.028	18.121	1.368-239.948
CD8^+^CD28^+^ (<14.55 vs. ≥14.55)	0.541	0.500	0.054-4.623			
CD8^+^PD-1^+^ (>41 vs. ≤41)	0.283	3.300	0.374-29.147			
CD4^+^CD28^+^ (≥48.93 vs. <48.93)	1.000	0.000	0.000-			

*The multivariate Logistic regression analysis was performed using the backward likelihood ratio (LR) method.

**Figure 4:**
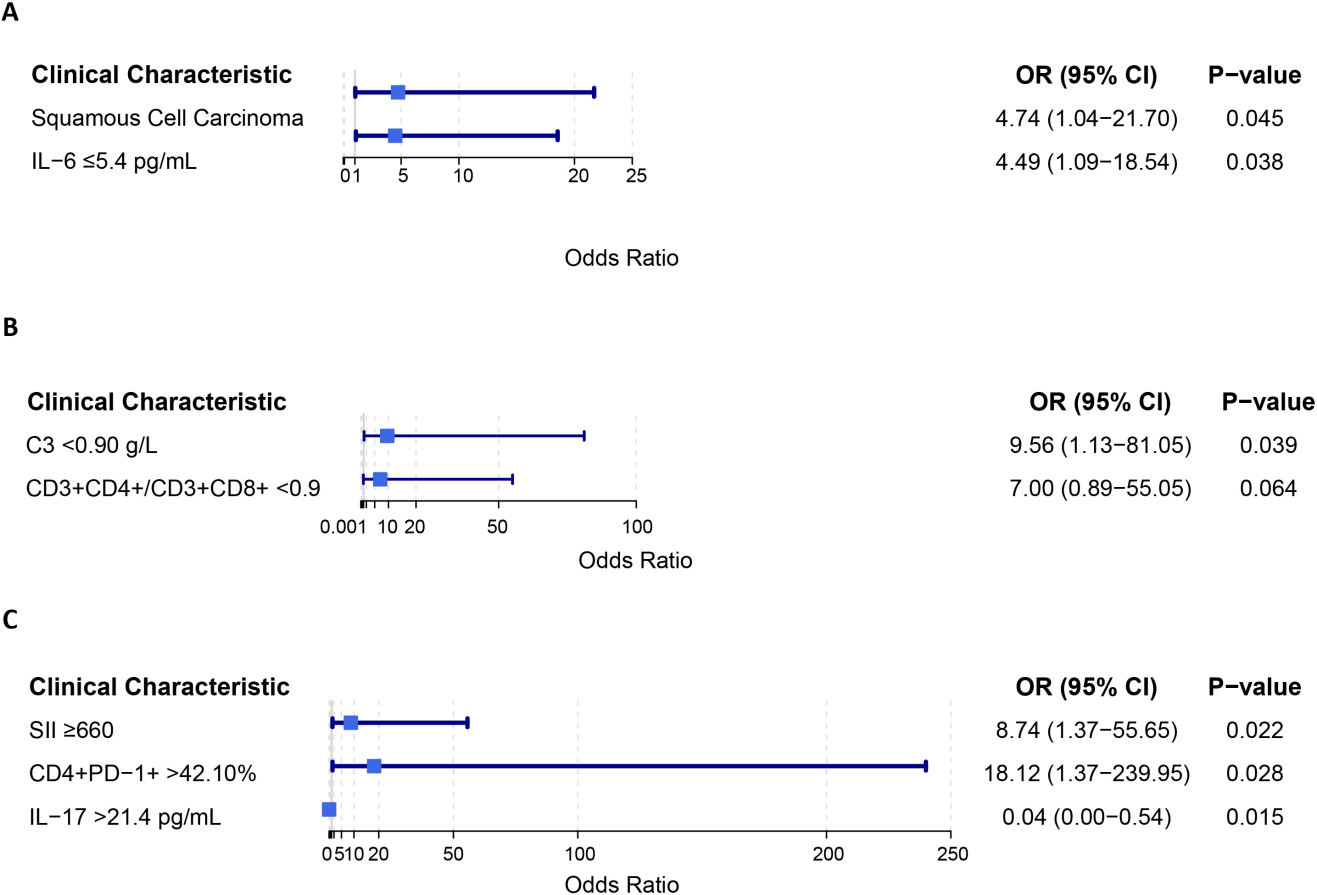
Forest plots of factors associated with efficacy and safety in multivariable analysis. **(A)** Factors independently associated with objective response. **(B)** Factors independently associated with treatment-emergent adverse events. **(C)** Factors independently associated with immune-related skin and subcutaneous tissue disorders.

To present the data more intuitively, a forest plot was generated to display the elements with P < 0.05 from the aforementioned multivariate logistic regression analyses (**Figure 4**).

## 4 Discussion

The prospective design of this study, with standardized treatment protocols and scheduled assessments, provides real-world evidence complementary to clinical trials. While not randomized, the active monitoring of consecutively enrolled patients enhances data reliability compared to purely retrospective analyses. The findings of this study demonstrate that cadonilimab exhibits favorable short-term efficacy and a manageable safety profile in women with cervical cancer. Patients achieved an ORR of 72.5% and a DCR of 90.2% upon completion of two cycles of cadonilimab treatment (10 mg/kg, Q3W). At the final follow-up cutoff date of December 31, 2025, 32 patients had experienced PD, resulting in a DCR of 37.3%. Kaplan-Meier analysis estimated a mPFS of 7.0 months (IQR: 4.0-10.0). Squamous cell carcinoma histology was identified as an independent predictor for higher ORR, whereas baseline IL-6 levels ≤5.4 pg/mL were independently associated with an increased likelihood of objective response, indicating a protective association. Furthermore, low baseline complement C3 levels were associated with the occurrence of any-grade adverse events during treatment, while a low CD3^+^CD4^+^/CD3^+^CD8^+^ ratio showed a similar but statistically non-significant trend. Further analysis focusing on irAEs revealed that baseline SII ≥660 and CD4^+^PD-1^+^ >42.10% were significant risk factors for the development of immune-related skin and subcutaneous tissue disorders. In contrast, baseline IL-17 >21.4 pg/mL was significantly associated with a reduced risk of these disorders.

The findings of this study are consistent with the results of a previously published phase II clinical trial (12), which evaluated the safety and preliminary efficacy of cadonilimab combined with chemotherapy with or without bevacizumab as first-line treatment for recurrent or metastatic cervical cancer. That trial demonstrated promising efficacy, with an ORR of 79.3% for cadonilimab (10 mg/kg, every three weeks) combined with chemotherapy with or without bevacizumab in first-line treatment, including an ORR of 75.0% in PD-L1 negative individuals. Another phase II trial (13) evaluating cadonilimab combined with chemotherapy as induction therapy for locally advanced cervical cancer also reported encouraging antitumor activity, achieving an ORR of 93.1% within just two weeks of treatment initiation. Real-world evidence further supports the clinical utility of cadonilimab, indicating that it may offer superior ORR compared to anti-PD-1 inhibitors for patients with recurrent or metastatic cervical cancer. This advantage appears particularly pronounced for patients who have previously failed anti-PD-1 therapy and those with PD-L1 negative tumors. Additionally, another real-world study was the first to evaluate the efficacy of cadonilimab combined with anti-angiogenic therapy in patients with recurrent or metastatic cervical cancer who had received multiple prior lines of therapy. This combination regimen demonstrated promising efficacy and an acceptable safety profile, with an ORR of 21.1%, a DCR of 42.1%, and a mPFS of 10.5 months. Collectively, these data suggest that cadonilimab exhibits therapeutic potential across the entire spectrum of cervical cancer management, from locally advanced to metastatic disease. It offers an important clinical option, especially for populations with suboptimal responses to conventional immunotherapy. Furthermore, the rapid and significant tumor shrinkage induced by cadonilimab is particularly critical in the neoadjuvant setting, where it may translate into the greatest overall survival benefit. This suggests that cadonilimab combined with chemotherapy and anti-angiogenic therapy holds promise as a preferred neoadjuvant regimen for locally advanced cervical cancer.

The logistic regression analysis in this study demonstrated that squamous cell carcinoma histology serves as an independent predictive factor for ORR. Compared to non-SCC patients (adenocarcinoma: 10/11 cases, 90.9%), SCC patients exhibited significantly better short-term therapeutic responses to cadonilimab treatment. This finding aligns with the results from another, larger real-world study comparing cadonilimab with anti-PD-1 inhibitors in recurrent or metastatic cervical cancer, which also identified squamous cell carcinoma as the sole factor independently associated with PFS and as an independent predictor for prolonged PFS in patients with R/M CC (p=0.010) (9). This positive association between squamous pathology and enhanced response to ICIs in cervical cancer is consistent with well-established observations in other solid tumors, such as non-small cell lung cancer and head and neck squamous cell carcinoma, where SCC histology is generally associated with greater sensitivity to immunotherapy (14, 15). The underlying mechanisms may involve several aspects: First, there are notable differences in tumor microenvironment characteristics. Research indicates that SCC tissues show significantly higher infiltration of immune effector cells, including CD4^+^ T cells and dendritic cells, compared to adenocarcinoma. This immune cell-enriched microenvironment may enhance the immune-activating effects of PD-1/CTLA-4 bispecific antibodies (16). Second, differential PD-L1 expression patterns exist, with SCC patients demonstrating higher PD-L1 positivity rates (CPS ≥1, approximately 60-80%) compared to adenocarcinoma (30-50%), which may influence the therapeutic efficacy of immune checkpoint inhibitors (17, 18). Furthermore, HPV-related immunogenicity features may represent another important influencing factor. Current evidence suggests that SCC is primarily driven by high-risk HPV infections (e.g., HPV16/18), whose expressed E6/E7 oncoproteins can serve as specific neoantigens that effectively activate T cell-mediated anti-tumor immune responses. In contrast, adenocarcinoma shows lower HPV infection rates (approximately 50-70%) and is more likely to develop through HPV-independent oncogenic pathways (such as HER2 amplification or PI3K mutations), potentially resulting in relatively weaker responses to immunotherapy (19–21). Notably, SCC typically exhibits a higher tumor mutational burden (TMB), which may generate more tumor neoantigens recognizable by the immune system. However, the precise mechanisms underlying the differential immune responses between SCC and adenocarcinoma in cervical cancer-particularly the potential influences of tumor immune infiltration patterns, driver gene mutation profiles, and HPV subtype distributions-require further elucidation through more in-depth research. We recommend future prospective studies incorporating multi-omics analyses (including genomics, transcriptomics, and immune microenvironment profiling) to validate current findings and provide more reliable evidence for developing personalized immunotherapy strategies.

Multivariable logistic regression analysis further confirmed that a baseline interleukin-6 (IL-6) level of ≤5.4 pg/mL was an independent protective factor associated with a higher ORR to cadonilimab. This finding aligns with the established consensus that elevated IL-6 is a poor prognostic factor across various malignancies, including cervical cancer. Mechanistically, as a key pro-inflammatory cytokine, elevated IL-6 can foster an immunosuppressive tumor microenvironment by activating the JAK/STAT3 pathway and expanding populations of myeloid-derived suppressor cells (MDSCs) and regulatory T cells (Tregs), which in turn suppress CD8^+^ T-cell function (22–24). These effects may attenuate the therapeutic efficacy of immune checkpoint inhibitors, including PD-1/CTLA-4 bispecific antibodies. Consequently, lower baseline IL-6 levels, as identified in our study, are associated with a more favorable tumor immune microenvironment and enhanced treatment response. While previous studies have suggested that inhibiting IL-6 signaling may enhance responses to immune checkpoint inhibitors in other cancer types (25–27), the current study extends these observations by demonstrating the potential utility of baseline IL-6 as a predictive biomarker in a real-world cohort of cervical cancer patients receiving cadonilimab.

It is important to clarify that the IL-6 cutoff of 5.4 pg/mL was predefined based on the upper limit of the normal reference range provided by the institutional clinical laboratory (i.e., normal value defined as IL-6 ≤5.4 pg/mL). This approach was used to categorize patients into “normal” versus “elevated” groups according to a clinically established local standard, rather than to derive a statistically optimized predictive threshold. Therefore, the results should be interpreted as demonstrating an association between clinically defined normal IL-6 levels and superior treatment response within this cohort, not as establishing a threshold with universal predictive value. The selection of this cutoff may be influenced by population-specific characteristics and assay methodology. Future prospective studies are warranted to validate this association, to explore the predictive value of dynamically monitoring IL-6 levels, and to investigate the potential for integrating IL-6 with other biomarkers (e.g., PD-L1 expression, tumor mutational burden) into multimodal predictive models.

This study confirms that cadonilimab demonstrates a manageable safety profile in patients with cervical cancer. Although the overall incidence of treatment-related adverse events was high (90.2%, n=46), with a notably early median time to first onset of 1.0 months, the majority were grade 1–2 and could be effectively controlled with clinical intervention. By system organ class, hematological toxicities were the most common (74.5%, n=38), followed by liver function-related events (39.2%, n=20) and immune-related skin and subcutaneous tissue disorders (23.5%, n=12). The incidence of grade ≥3 adverse events was relatively low (31.4%, n=16), with a later median time to first onset of 8.0 months.

While previous studies (7, 12, 13) have identified anemia, leukopenia, and neutropenia as common AEs associated with cadonilimab in cervical cancer, the hematological toxicity rate observed in our cohort (74.5%) was substantially higher than that reported for cadonilimab monotherapy. This discrepancy strongly suggests that the observed myelosuppression is more likely attributable to the concurrent chemotherapy backbone rather than the immune checkpoint inhibitor component. Although univariate logistic regression analysis of “any AEs” (including hematological toxicities) did not show statistical significance for concomitant chemotherapy (**Table 7**), this finding should be interpreted with caution due to several methodological limitations. These include the relatively small sample size, substantial group imbalance (46 patients [90.2%] received cadonilimab plus chemotherapy ± bevacizumab vs. only 5 patients [9.8%] receiving cadonilimab monotherapy), and potential confounding by indication. These factors collectively limit statistical power and may obscure the contribution of chemotherapy to hematological toxicity. Future studies with larger, more balanced cohorts are warranted to better delineate the respective contributions of chemotherapy and immunotherapy to AE profiles in cadonilimab-based regimens.

**Table 7:** Logistic regression analysis of concomitant chemotherapy on AEs.

Adverse event type	With chemo (n=46)	Without chemo (n=5)	OR (95% CI)	P value
Any adverse event	41 (89.1)	5 (100.0)	0.600 (0.057-6.312)	0.671
Hematologic adverse event	34 (73.9)	4 (80.0)	0.833 (0.141-4.922)	0.841
Skin and subcutaneous tissue disorders	10 (21.7)	2 (40.0)	1.360 (0.228-8.105)	0.736

Notably, the overall incidence of grade ≥3 immune-related adverse events in this study was lower than that reported in previous studies of PD-1 antibody combined with chemotherapy or PD-1/CTLA-4 bispecific antibody combined with chemotherapy in cervical cancer (9, 28, 29). Specifically, several severe irAEs commonly observed in other ICI-plus-chemotherapy studies, such as high-grade rash, colitis, pneumonitis, endocrinopathies, and central nervous system toxicities, occurred at very low rates or were not reported in the present analysis. Several methodological factors in our real-world study should be considered when interpreting these lower reported rates. First, mild or subclinical irAEs may have been under-ascertained because specific diagnostic tests (e.g., thyroid function tests, pulmonary imaging for pneumonitis) were not routinely performed in the absence of clinical suspicion due to cost and feasibility constraints in standard practice. Second, the diagnostic discrimination between irAEs and other common conditions (e.g., infection-related pneumonia or colitis) can be challenging, potentially leading to under-reporting. In contrast, adverse events such as hematological toxicities, liver function abnormalities (e.g., elevated AST/ALT), and skin/subcutaneous tissue disorders are routinely monitored through standard laboratory tests and clinical examinations at each treatment visit. Therefore, their reported incidence in our study is less susceptible to the aforementioned ascertainment biases and remains comparable to rates observed in similar studies, with liver function abnormalities being the most common non-hematological irAE. This overall safety trend aligns with findings from prior phase II/III trials of cadonilimab combined with chemotherapy with or without bevacizumab (30), suggesting that its bispecific design may help mitigate the risk of certain organ-specific irAEs, particularly cutaneous and gastrointestinal toxicities. However, given the relatively short median follow-up of only 11 months in this study, which further limits the cumulative observation period for late-onset irAEs, the cumulative incidence of irAEs may be influenced by the observation period. Longer follow-up is needed to further evaluate long-term safety.

In summary, the present study supports the favorable and manageable risk profile of cadonilimab in clinical practice, while underscoring the importance of close monitoring of hematological parameters and liver function, particularly in patients with pre-existing cytopenia or hepatic impairment at baseline.

This study employed logistic regression analysis to investigate factors associated with the occurrence of AEs in cervical cancer patients treated with cadonilimab. The analysis revealed that a baseline complement C3 level <0.90 g/L was a significant risk factor for AEs. Furthermore, we observed a trend toward a higher incidence of AEs in patients with a lower baseline CD3^+^CD4^+^/CD3^+^CD8^+^ ratio during cadonilimab treatment, although this trend did not reach statistical significance (p > 0.05). These findings suggest potential associations between baseline immunology-related biomarker levels and cadonilimab safety. Further exploration specifically into irAEs affecting the skin and subcutaneous tissue revealed distinct predictive profiles. Multivariate analysis identified that elevated baseline levels of certain markers, including a SII ≥660 and CD4^+^PD-1^+^ >42.10%, were independent risk factors for these irAEs. Conversely, a higher baseline IL-17 level (>21.4 pg/mL) was independently associated with a markedly reduced risk, indicating a potential protective effect.

In the analysis of irAEs, a baseline Systemic Immune-Inflammation Index (SII) ≥660 was identified as an independent risk factor for immune-related disorders of the skin and subcutaneous tissue (OR = 8.742, 95% CI = 1.372–55.648, P = 0.022). The Systemic Immune-Inflammation Index (31) is a novel inflammatory biomarker calculated as platelet count multiplied by neutrophil count divided by lymphocyte count. This composite index dynamically reflects changes in neutrophil, platelet, and lymphocyte counts, serving as an integrated marker to assess systemic inflammatory status, pro-thrombotic tendency, and immune function. A higher baseline SII suggests a pre-existing microenvironment characterized by neutrophilia, potentially accompanied by the infiltration of pro-inflammatory subsets or myeloid-derived suppressor cells, platelet activation, and relative lymphocytopenia before treatment initiation. This pro-inflammatory state, coupled with impaired immune surveillance, may predispose the immune system to a pre-activated condition, thereby lowering the threshold for excessive immune activation upon dual blockade of the PD-1 and CTLA-4 pathways by cadonilimab (32–34). The consequent amplification of the inflammatory cascade, which may include enhanced cytokine release and increased recruitment of immune cells to skin tissues, could disrupt the local immune tolerance balance and ultimately trigger an autoimmune response against cutaneous antigens.

Baseline interleukin-17 (IL-17) levels >21.4 pg/mL were identified as an independent protective factor against immune-related skin and subcutaneous tissue disorders (OR = 0.042, 95% CI = 0.003–0.542, P = 0.015). Conventionally, IL-17, a key pro−inflammatory cytokine primarily produced by Th17 cells, is regarded as central to cutaneous barrier defense and inflammatory responses (35). It promotes the release of antimicrobial peptides (e.g., β-defensins), chemokines (e.g., CXCL1, CXCL8), and various pro-inflammatory mediators (e.g., IL-6, TNF-α) from keratinocytes, thereby recruiting and activating neutrophils. This mechanism would, in theory, be expected to increase the risk of irAEs. However, emerging research suggests that the development of irAEs is linked not only to the activation of inflammatory pathways but, more critically, to the disruption of immune homeostasis. For instance, Nina Flatt and colleagues observed that while a higher baseline IL-7 level might suggest an increased risk of irAEs, a decrease in IL-7 levels early during treatment held greater predictive value, indicating that a disturbance in immune homeostasis is pivotal for irAE onset (36). In the context of our study, a higher baseline IL-17 level may represent a pre-treatment immunological state in some patients characterized by “moderate activity and self-balance.” Such a state could potentially help maintain immunoregulatory functions following immune checkpoint inhibitor therapy, thereby buffering against tissue damage from excessive immune activation and reducing the risk of cutaneous irAEs.

Additionally, this study found that a higher pretreatment proportion of CD4^+^PD-1^+^ T cells (>42.10%) in peripheral blood was independently associated with the subsequent development of immune-related skin and subcutaneous tissue disorders (OR = 18.121, 95% CI: 1.368–239.948, P = 0.028). CD4^+^ T cells are central regulators of adaptive immunity, and the upregulation of programmed death receptor-1 (PD-1) on their surface typically occurs under conditions of chronic antigen exposure or persistent immune activation. A high baseline CD4^+^PD-1^+^ level may therefore reflect a state of systemic immune pre-activation or chronic inflammation in the patient prior to treatment, potentially driven by factors such as persistent tumor antigen stimulation, latent viral infection (e.g., HPV), or an autoimmune background. When the PD-1 pathway is blocked by cadonilimab, these “primed” CD4^+^ T cells may be rapidly disinhibited, leading to excessive proliferation and cytokine release, which could subsequently attack skin tissues sharing common antigens and trigger cutaneous irAEs. This observation is consistent with findings from other clinical studies. For instance, research in melanoma patients has associated a higher baseline proportion of CD4^+^ naïve T cells with the occurrence of severe irAEs, suggesting that a pre-activated immune state may predispose to irAE development (37). Another study found that different pretreatment T cell subsets (e.g., Th2, Th17) in peripheral blood were closely associated with irAEs in specific organs, further confirming the predictive role of baseline immune status for irAE types (38). Furthermore, a Phase Ib trial (39) demonstrated that higher baseline levels of CD4^+^PD-1^+^OX40^+^ T cells were associated with better clinical outcomes, indirectly reflecting the link between this cell population and a state of immune system activation.

This study has several inherent limitations that should be considered when interpreting the findings. As a single-center observational study with a relatively small sample size (N = 51), the results are subject to potential selection bias and limited generalizability. The modest number of events, particularly the low incidence of certain immune-related adverse events, contributed to statistical instability in multivariable analyses, as reflected in wide confidence intervals. Accordingly, all biomarker findings should be regarded as exploratory and hypothesis-generating, warranting validation in larger cohorts. Inclusion criteria requiring at least two cycles of cadonilimab and one post-baseline assessment may have introduced selection bias by excluding patients with early progression or toxicity-related discontinuation, potentially overestimating efficacy and underestimating early adverse events. Treatment heterogeneity, which included triple, double, and monotherapy regimens, along with the absence of randomization or adjustment for confounding by indication, complicates attribution of effects specifically to cadonilimab. Moreover, the small size of the monotherapy group (n=5) precludes meaningful comparative analysis. The median follow-up of 11 months limits assessment of long-term outcomes and late-onset toxicities, making this an interim analysis. The observed lower incidence of certain irAEs (e.g., pneumonitis, colitis, endocrinopathies) compared with prior trials may reflect under-ascertainment due to less intensive routine screening in real-world practice, rather than a definitively superior safety profile. PD-L1 expression status was missing for 64.7% of patients and could not be incorporated into analyses, limiting the interpretability of findings in relation to current treatment paradigms. The biomarker cutoff for SII was derived from a data-driven ROC analysis, which introduces a risk of overfitting; therefore, this finding requires independent validation. Although the IL-6 cutoff was predefined based on the laboratory normal range, its predictive utility remains uncertain and should not be considered universally applicable. No adjustment was made for multiple comparisons in exploratory analyses, raising the possibility that some significant findings may be due to chance. Finally, as an observational study without a control group, causal inferences regarding the comparative effectiveness of cadonilimab versus other treatments cannot be made, and historical comparisons with clinical trial data should be interpreted cautiously.

Given these limitations, the findings of this study should be considered hypothesis-generating and require validation in larger, prospective settings. Future research should prioritize prospective, multicenter trials to confirm the efficacy of cadonilimab, validate the predictive value of potential biomarkers, such as those related to complement pathway involvement in immune regulation, and explore rational combination strategies with chemoradiotherapy or other immunotherapies. Furthermore, extended follow-up of current and future cohorts, combined with the deliberate inclusion of underrepresented patient subgroups (e.g., those with adenocarcinoma or mixed histology), is critical to fully delineate the long-term clinical potential and optimal therapeutic positioning of cadonilimab.

## 5 Conclusions

In this observational cohort study, cadonilimab demonstrated encouraging short-term objective response rates with a manageable safety profile in patients with cervical cancer. Logistic regression analysis identified squamous cell carcinoma histology as an independent factor associated with higher objective response rate, while lower baseline IL-6 levels (≤5.4 pg/mL, defined by institutional normal range) were independently associated with increased probability of response. Furthermore, the occurrence of immune-related adverse events involving the skin and subcutaneous tissue was associated with elevated baseline levels of specific immunologic markers: SII ≥660 and CD4+PD-1+ >42.10%, while higher baseline IL-17 (>21.4 pg/mL) was identified as a protective factor. However, due to the small sample size, limited number of events, and wide confidence intervals, these biomarker findings should be considered preliminary and hypothesis-generating, not definitive predictive markers. They require validation in larger, prospective studies with standardized biomarker assays and longer follow-up before consideration for clinical application.

## Data Availability

The original contributions presented in the study are included in the article/supplementary material. Further inquiries can be directed to the corresponding author.

## References

[1] Bray F Laversanne M Sung H Ferlay J Siegel RL Soerjomataram I . Global cancer statistics 2022: GLOBOCAN estimates of incidence and mortality worldwide for 36 cancers in 185 countries. CA Cancer J Clin. (2024) 74(3):229–63. doi: 10.3322/caac.21834. PMID: 38572751

[2] Bruni L Serrano B Roura E Alemany L Cowan M Herrero R . Cervical cancer screening programmes and age-specific coverage estimates for 202 countries and territories worldwide: a review and synthetic analysis. Lancet Glob Health. (2022) 10(8):e1115–27. doi: 10.1016/S2214-109X(22)00241-8. PMID: 35839811 PMC9296658

[3] Momenimovahed Z Mazidimoradi A Maroofi P Allahqoli L Salehiniya H Alkatout I . Global, regional and national burden, incidence, and mortality of cervical cancer. Cancer Rep. (2023) 6(3):e1756. doi: 10.1002/cnr2.1756. PMID: 36545760 PMC10026270

[4] Monk BJ Colombo N Tewari KS Dubot C Caceres MV Hasegawa K . First-line pembrolizumab + chemotherapy versus placebo + chemotherapy for persistent, recurrent, or metastatic cervical cancer: final overall survival results of KEYNOTE-826. J Clin Oncol. (2023) 41(36):5505–11. doi: 10.1200/JCO.23.00914. PMID: 37910822

[5] O'Malley DM Neffa M Monk BJ Melkadze T Huang M Kryzhanivska A . Dual PD-1 and CTLA-4 checkpoint blockade using balstilimab and zalifrelimab combination as second-line treatment for advanced cervical cancer: an open-label phase II study. J Clin Oncol. (2022) 40(7):762–71. doi: 10.1200/JCO.21.02067, PMID: 34932394 PMC8887945

[6] Oaknin A Moore KN Meyer T González JL Devriese L Amin A . 520MO Safety and efficacy of nivolumab (NIVO) ± ipilimumab (IPI) in patients (pts) with recurrent/metastatic cervical cancer (R/M Cx Ca) in checkmate 358. Ann Oncol. (2022) 33:S782. doi: 10.1016/j.annonc.2022.07.648, PMID: 38826717

[7] Wu X Sun Y Yang H Wang J Lou H Li D . Cadonilimab plus platinum-based chemotherapy with or without bevacizumab as first-line treatment for persistent, recurrent, or metastatic cervical cancer (COMPASSION-16): a randomised, double-blind, placebo-controlled phase 3 trial in China. Lancet. (2024) 404(10463):1668–76. doi: 10.1016/S0140-6736(24)02135-4. PMID: 39426385

[8] Keam SJ . Cadonilimab: first approval. Drugs. (2022) 82(12):1333–9. doi: 10.1007/s40265-022-01761-9. PMID: 35986837

[9] Pan B Huang H Wan T Huang Q He S Xu S . The comparison of efficacy and safety between cadonilimab (PD-1/CTLA-4) and anti-PD-1 inhibitors in patients with recurrent or metastatic cervical cancer: a retrospective real-world study. Front Immunol. (2025) 16:1582299. doi: 10.3389/fimmu.2025.1582299. PMID: 40529375 PMC12171111

[10] Zhou Y Shi J Stein R Liu X Baldassano RN Forrest CB . Missing data matter: an empirical evaluation of the impacts of missing EHR data in comparative effectiveness research. J Am Med Inform Assoc. (2023) 30(7):1246–56. doi: 10.1093/jamia/ocad066. PMID: 37337922 PMC10280351

[11] Barzi F Woodward M . Imputations of missing values in practice: results from imputations of serum cholesterol in 28 cohort studies. Am J Epidemiol. (2004) 160(1):34–45. doi: 10.1093/aje/kwh175. PMID: 15229115

[12] Lou H Cai H Huang X Li G Wang L Liu F . Cadonilimab combined with chemotherapy with or without bevacizumab as first-line treatment in recurrent or metastatic cervical cancer (COMPASSION-13): a phase 2 study. Clin Cancer Res. (2024) 30(8):1501–8. doi: 10.1158/1078-0432.CCR-23-3162. PMID: 38372727 PMC11016896

[13] Ding L He S Yang J Rao J Ren Y Xiao L . Induction cadonilimab combined with chemotherapy followed by chemoradiotherapy for locally advanced cervical cancer: A multicenter, single-arm, phase II trial. J Clin Oncol. (2025) 43:5530. doi: 10.1200/JCO.2025.43.16_suppl.5530, PMID: 41909186

[14] Li F Zhai S Lv Z Yuan L Wang S Jin D . Effect of histology on the efficacy of immune checkpoint inhibitors in advanced non-small cell lung cancer: A systematic review and meta-analysis. Front Oncol. (2022) 12:968517. doi: 10.3389/fonc.2022.968517. PMID: 36439448 PMC9685340

[15] van Duijvenvoorde M Derks S Bahce I Leemans CR van de Ven R Fransen MF . Comparison of the tumor microenvironments of squamous cell carcinoma at different anatomical locations within the upper aerodigestive tract in relation to response to ICI therapy. Clin Transl Immunol. (2022) 11(1):e1363. doi: 10.1002/cti2.1363. PMID: 35035956 PMC8747970

[16] Li J Xue X Zhang Y Ding F Wu W Liu C . The differences in immune features and genomic profiling between squamous cell carcinoma and adenocarcinoma - A multi-center study in Chinese patients with uterine cervical cancer. Gynecol Oncol. (2023) 175:133–41. doi: 10.1016/j.ygyno.2023.05.071. PMID: 37356314

[17] Huang RS Haberberger J Murugesan K Danziger N Hiemenz M Severson E . Clinicopathologic and genomic characterization of PD-L1-positive uterine cervical carcinoma. Mod Pathol. (2021) 34(7):1425–33. doi: 10.1038/s41379-021-00780-3. PMID: 33637877

[18] Baek MH Chen L Tekin C Cristescu R Jin XY Shao C . Prevalence and prognostic value of PD-L1 expression and tumor mutational burden in persistent, recurrent, or metastatic cervical cancer. J Gynecol Oncol. (2024) 35(6):e105. doi: 10.3802/jgo.2024.35.e105. PMID: 38857910 PMC11543264

[19] Giorgi Rossi P Carozzi F Ronco G Allia E Bisanzi S Gillio-Tos A . p16/ki67 and E6/E7 mRNA accuracy and prognostic value in triaging HPV DNA-positive women. J Natl Cancer Inst. (2021) 113(3):292–300. doi: 10.1093/jnci/djaa105. PMID: 32745170 PMC7936054

[20] Peng S Ferrall L Gaillard S Wang C Chi WY Huang CH . Development of DNA vaccine targeting E6 and E7 proteins of human papillomavirus 16 (HPV16) and HPV18 for immunotherapy in combination with recombinant vaccinia boost and PD-1 antibody. MBio. (2021) 12(1):10–1128. doi: 10.1128/mBio.03224-20. PMID: 33468698 PMC7845631

[21] Kuno I Takayanagi D Asami Y Murakami N Matsuda M Shimada Y . TP53 mutants and non-HPV16/18 genotypes are poor prognostic factors for concurrent chemoradiotherapy in locally advanced cervical cancer. Sci Rep. (2021) 11(1):19261. doi: 10.1038/s41598-021-98527-2. PMID: 34584128 PMC8478905

[22] Thuya WL Cao Y Ho PC-L Wong AL-A Wang L Zhou J . Insights into IL-6/JAK/STAT3 signaling in the tumor microenvironment: Implications for cancer therapy. Cytokine Growth Factor Rev. (2025) 85:26–42. doi: 10.1016/j.cytogfr.2025.01.003, PMID: 39893129 PMC13560456

[23] Tengesdal IW Dinarello A Powers NE Burchill MA Joosten LA Marchetti C . Tumor NLRP3-derived IL-1β drives the IL-6/STAT3 axis resulting in sustained MDSC-mediated immunosuppression. Front Immunol. (2021) 12:661323. doi: 10.3389/fimmu.2021.661323. PMID: 34531850 PMC8438323

[24] Jeong H Koh J Kim S Yim J Song SG Kim H . Cell-intrinsic PD-L1 signaling drives immunosuppression by myeloid-derived suppressor cells through IL-6/Jak/Stat3 in PD-L1-high lung cancer. J Immunother Cancer. (2025) 13(3):e010612. doi: 10.1136/jitc-2024-010612. PMID: 40050048 PMC11887297

[25] Arends R Guo X Baverel PG González-García I Xie J Morsli N . Association of circulating protein biomarkers with clinical outcomes of durvalumab in head and neck squamous cell carcinoma. Oncoimmunology. (2021) 10(1):1898104. doi: 10.1080/2162402X.2021.1898104. PMID: 33796405 PMC7993189

[26] Laino AS Woods D Vassallo M Qian X Tang H Wind-Rotolo M . Serum interleukin-6 and C-reactive protein are associated with survival in melanoma patients receiving immune checkpoint inhibition. J Immunother Cancer. (2020) 8(1):e000842. doi: 10.1136/jitc-2020-000842. PMID: 32581042 PMC7312339

[27] Keegan A Ricciuti B Garden P Cohen L Nishihara R Adeni A . Plasma IL-6 changes correlate to PD-1 inhibitor responses in NSCLC. J Immunother Cancer. (2020) 8(2):e000678. doi: 10.1136/jitc-2020-000678. PMID: 33020238 PMC7537334

[28] Kusuma F Glenardi G Mangkuliguna G Winarto H Purwoto G Utami TW . Efficacy, safety, and patient-reported outcome of immune checkpoint inhibitor in gynecologic cancers: A systematic review and meta-analysis of randomized controlled trials. PLoS One. (2024) 19(8):e0307800. doi: 10.1371/journal.pone.0307800. PMID: 39133693 PMC11318932

[29] Liu Y Wu L Tong R Yang F Yin L Li M . PD-1/PD-L1 inhibitors in cervical cancer. Front Pharmacol. (2019) 10:65. doi: 10.3389/fphar.2019.00065. PMID: 30774597 PMC6367228

[30] Ma W Li Y Lu Y Liang Z Yu H Han J . A multicenter retrospective study: Impact of first-line treatment strategies on second-line efficacy and safety of regorafenib with or without PD-1 inhibitors in unresectable hepatocellular carcinoma. J Hepatocell Carcinoma. (2025) 12:2123–37. doi: 10.2147/JHC.S456712. PMID: 40980778 PMC12449871

[31] Kou J Huang J Li J Wu Z Ni L . Systemic immune-inflammation index predicts prognosis and responsiveness to immunotherapy in cancer patients: a systematic review and meta-analysis. Clin Exp Med. (2023) 23(8):3895–905. doi: 10.1007/s10238-023-01035-y. PMID: 36966477

[32] Zheng F Meng Q Zhang L Chen J Zhao L Zhou Z . Prognostic roles of hematological indicators for the efficacy and prognosis of immune checkpoint inhibitors in patients with advanced tumors: a retrospective cohort study. World J Surg Oncol. (2023) 21(1):198. doi: 10.1186/s12957-023-03077-8. PMID: 37420219 PMC10326931

[33] Chen Q Zhai B Li J Wang H Liu Z Shi R . Systemic immune-inflammatory index predict short-term outcome in recurrent/metastatic and locally advanced cervical cancer patients treated with PD-1 inhibitor. Sci Rep. (2024) 14(1):31528. doi: 10.1038/s41598-024-82976-6. PMID: 39732889 PMC11682050

[34] Zhan Q Xu N Fan J Wang J Li F . Prognostic value of systemic immune-inflammation index in cervical cancer: a systematic review and meta-analysis. J Obstet Gynaecol. (2025) 45(1):2601171. doi: 10.1080/01443615.2025.2601171. PMID: 41378768

[35] Mills KHG . IL-17 and IL-17-producing cells in protection versus pathology. Nat Rev Immunol. (2023) 23(1):38–54. doi: 10.1038/s41577-022-00746-9. PMID: 35790881 PMC9255545

[36] Flatt N Walter A Kochanek C Beikirch M DeTemple VK Angela Y . A composite score of serum cytokines enables early identification of patients at high risk for irAEs under immune checkpoint inhibition. Front Immunol. (2025) 16:1733357. doi: 10.3389/fimmu.2025.1733357. PMID: 41394871 PMC12696155

[37] Kovacsovics-Bankowski M Sweere JM Healy CP Sigal N Cheng LC Chronister WD . Lower frequencies of circulating suppressive regulatory T cells and higher frequencies of CD4+ naïve T cells at baseline are associated with severe immune-related adverse events in immune checkpoint inhibitor-treated melanoma. J Immunother Cancer. (2024) 12(1):e008056. doi: 10.1136/jitc-2023-008056. PMID: 38233101 PMC10806651

[38] Bukhari S Henick BS Winchester RJ Lerrer S Adam K Gartshteyn Y . Single-cell RNA sequencing reveals distinct T cell populations in immune-related adverse events of checkpoint inhibitors. Cell Rep Med. (2023) 4(1):100868. doi: 10.1016/j.xcrm.2022.100868. PMID: 36513074 PMC9873824

[39] Baldini C Danlos FX Varga A Texier M Halse H Mouraud S . Safety, recommended dose, efficacy and immune correlates for nintedanib in combination with pembrolizumab in patients with advanced cancers. J Exp Clin Cancer Res. (2022) 41(1):217. doi: 10.1186/s13046-022-02423-0. PMID: 35794623 PMC9260998

